# The First Reported Case of a Child with Two Different Rare Metabolic Disorders: Very Long-Chain Acyl-CoA Dehydrogenase Deficiency and Encephalomyopathic Mitochondrial DNA Depletion Syndrome 13

**DOI:** 10.1055/s-0043-1775979

**Published:** 2023-10-10

**Authors:** Maha Alotaibi, Amal Alqasmi, Faisal Albassam, Turki Alkahtani, Muath Alqahtany, Mohammed Alkhaldi

**Affiliations:** 1 Department of Genetic, Children Hospital, King Saud Medical City, Riyadh, Saudi Arabia; 2 Department of Pediatric Neurology and Epilepsy, King Saud Medical City, Riyadh, Saudi Arabia; 3 Collage of Medicine, AlMaarefa University, Riyadh, Saudi Arabia

**Keywords:** FBXL4, hypoglycemia, mitochondrial DNA, mitochondrial diseases, very long-chain acyl-CoA dehydrogenase deficiency, pediatric genetics

## Abstract

One of the most common inborn errors in fatty acid β oxidation (FAO) is a very long-chain acyl-coenzyme A dehydrogenase (VLCAD) deficiency. It is autosomal recessive. The enzyme used in the first phase of FAO is VLCAD. The enzyme is responsible for β oxidation spiral pathway's initial step, the dehydrogenation process of long-chain fatty acyl-CoA. The phenotypes include hypoglycemia, hepatomegaly, cardiomyopathy, and occasionally abrupt mortality. Most VLCAD deficiencies in newborns are now detected during the neonatal period due to the development of newborn screening programs. Mitochondrial DNA depletion syndromes (MTDPS) are one of the rarest metabolic disorders. It is an autosomal recessive disease caused by defects in genes necessary for the maintenance of mitochondrial DNA (mtDNA). One of these *FBXL4* (F-box and leucine-rich repeat protein 4) variants causes encephalomyopathic mtDNA depletion syndrome 13 (MTDPS13), which presents as a failure to thrive, severe global developmental delay, hypotonia, early infantile onset of encephalopathy, and lactic acidosis. We report here the case of a Saudi infant born to consanguineous parents who presented to us with severe failure to thrive, profound neurodevelopmental delays, and facial dysmorphic features. Whole-exome sequencing (WES) showed the infants had MTDPS13. The FBXL4 variant c.1698A > G p. (Ile566Met) has previously been described as a disease that causes developmental delay and lactic acidosis, and another variant has also been detected in the patient. The ACADVL variant c.134C > A p. (Ser45*) has previously been described to cause VLCAD deficiency. A comprehensive literature review showed our patient to be the first case of MTDPS13 and VLCAD reported to date worldwide.

## Introduction

An inherited metabolic disorder (IMD) of fatty acid oxidation called very long-chain acyl-coenzyme A dehydrogenase (VLCAD; OMIM #201475) deficiency is brought on by pathogenic variants in the ACADVL gene. The first stage of mitochondrial oxidation of long-chain fatty acids with chain lengths of 14 to 20 carbons is catalyzed by the VLCAD enzyme. These long-chain fatty acids cannot be digested in VLCAD, which might result in metabolic crises brought on by an insufficient supply of energy.

There is a spectrum of VLCAD phenotypes based on clinical presentation, biochemical tests, and/or genetic analysis. Three subgroups of VLCAD are frequently distinguished: severe, moderate, and mild. 1 The severe, or early-onset, type of the disease often manifests within the first months of life and is characterized by cardiomyopathy, arrhythmias, hypotonia, hepatomegaly, and hypoglycemia. The mild type often manifests as periods of hypoketotic hypoglycemia and hepatomegaly linked to a catabolic load during late infancy or early adolescence. Compared with severe VLCAD, cardiomyopathy is substantially less likely in mild VLCAD. Usually beginning at adolescence, the mild or late variant is characterized by episodic myopathy coupled with exercise intolerance and rhabdomyolysis.

The second disorder found in our case is a rare autosomal recessive disorder called encephalomyopathic mtDNA depletion syndrome 13 (MTDPS13; OMIM # 615471), which is caused by biallelic variants in the FBXL4 gene (MIM 605654). The FBXL4 (F-box and leucine-rich repeat protein 4) gene has a crucial role in preserving the stability and integrity of mitochondrial DNA (mtDNA). Worldwide, different breeds are affected by defects in mtDNA maintenance by variants of the FBXL4 gene. Clinically, MTDPS13 often exhibits encephalopathy, hypotonia, failure to thrive, prolonged lactic acidosis, and developmental delays, 2 3 4 among others. More than half of the patients have feeding difficulties, and some show microcephaly and have characteristic facial features, for example, elongated, protruding ears, epicanthal folds, downslanting palpebral fissures, thick eyebrows, and cataracts. Neuroimaging often reveals white matter abnormalities. 5 To our knowledge, no cases of FBXL4 deficiency and VLCAD deficiency have been documented in the literature. Consequently, we chose to make public the first case of encephalomyopathic MTDPS13 associated with VLCAD deficiency.

## Methods

Peripheral blood samples were collected from the patient and his parents. Written consent was taken before the sample was collected. The exome of the index patient was sequenced at CENTOGENE (Rostock, Germany).

Genomic DNA is enzymatically fragmented, and the target regions are enriched using DNA capture probes. These regions include approximately 41 Mb of the human coding exome (targeting >98% of the coding RefSeq from the human genome build GRCh37/hg19), as well as the mitochondrial genome. The generated library is sequenced on an Illumina platform to obtain at least 20 times coverage depth for greater than 98% of the targeted bases. An in-house bioinformatics pipeline, including read alignment to the GRCh37/hg19 genome assembly and the revised Cambridge Reference Sequence (rCRS) of the Human Mitochondrial DNA (NC_012920), variant calling, annotation, and comprehensive variant filtering, is applied. All variants with a minor allele frequency (MAF) of less than 1% in the gnomAD database and disease-causing variants reported in HGMD, ClinVar, or CentoMD are evaluated. The investigation for relevant variants is focused on coding exons and flanking ± 10 intronic nucleotides of genes with clear gene-phenotype evidence (based on OMIM information). The American College of Medical Genetics and Genomics (ACMG) guidelines for classification of variants and all relevant variants related to the phenotype of the patient are reported.

## Clinical Description

A 33-day-old Saudi male infant was referred to a genetic physician as a case of severe failure to thrive, recurrent hypoglycemia, and positive newborn screening for VLCAD on special formula. He was born to consanguineous, healthy parents after a full-term, uneventful pregnancy. His birth weight, height, and head circumference were 2.10 kg (third percentile), 31.5 cm (third percentile), and 44.05 cm (50th percentile), respectively. His Apgar score was unknown. He looked dysmorphic with a small head, an elongated face, bitemporal narrowing, epicanthal folds, downslanting palpebral fissures, thick eyebrows, micrognathia, and a low-set ears. In his postnatal history, he was kept in the neonatal intensive care unit (NICU) due to poor sucking and severe hypoglycemia. He was managed accordingly and discharged home after 10 days with encouragement on a special formula and mitochondrial cocktails. The patient was readmitted 1 week ago with a history of lethargy, hypoglycemia, and persistent lactic acidosis. The patient was admitted to the ICU, and dichloroacetic acid and sodium bicarbonate were used to treat metabolic acidosis and hyperlactatemia. Dextrose 10%, riboflavin, thiamine, and biotin were added to rule out other possible causes of mitochondrial disorders. But his condition deteriorated. He needed mechanical ventilator support and he expired.

There was no family history of genetic or metabolic disease; there was no history of death, miscarriage, or brain atrophy. The patient had a healthy 4-year-old sister.

**Fig. 1:**
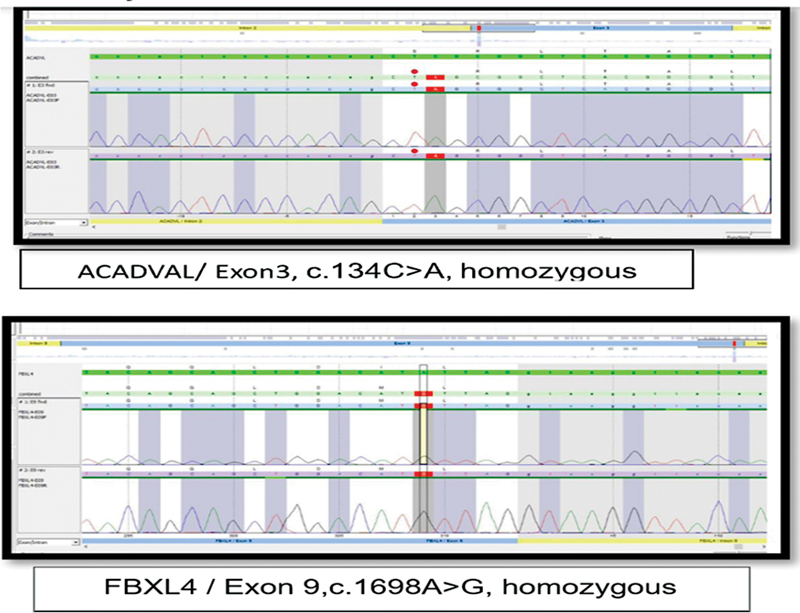
Chromatogram of the patient.

Laboratory investigations revealed venous blood gas with pH 7.37, pCO _2_ 1.5 mm Hg, and HCO _3_ 15.4 mmol/L, lactate of 6.9 mmol/L (normal 2 mmol/L), ammonia of 81.6 mol/L (normal 40 mmol/L), elevated alanine at 721 U/L (normal 41 U/L), and aspartate aminotransferase of 515.2 U/L (normal 40 U/L). The tandem mass spectrometry showed *significant elevation* of the *biochemical marker C14:1* (3.744 µmol/L), which indicated *VLCAD* , and urine analysis by gas chromatography/mass spectrometry (GC-MS) revealed significant lactic acid and acetoacetate levels that were all elevated (higher than the normal reference values). Chest radiography demonstrated an increased cardiothoracic ratio and mild cardiomegaly, and echocardiographic evaluation showed a small size atrial septal defect (ASD) with increased left ventricular thickness and normal systolic function in favor of hypertrophic cardiomyopathy. Transcranial ultrasound was unremarkable. Whole-exome sequencing detected two different variant variants: a homozygous pathogenic variant in ACADVL c.134C > A p. (Ser45*) and a homozygous pathogenic variant in the FBXL4 c.1698A > G p. (Ile566Me). Genetic testing and target variants were done for the parents, and genetic counseling was performed ( Fig. 1 ).

## Discussion

Clinical and genetic diversities characterize inherited metabolic diseases, which are rare disorders. Atypical features are usually described as an expansion of the established phenotype. In this study, we describe the case of a newborn from Saudi Arabia who had unique characteristics that were later explained by a specific combination of two metabolic disorders, MTDPS13 and VLCAD deficits, which led to a reduction in the availability of energy in the skeletal muscle or brain. In such cases, energy demand is raised, and both situations are known to be triggered by fasting, infection, and other catabolic circumstances.

In this report, we present a genetic case of two different diseases with an unusual phenotype and two different gene variants. This is an interesting presentation, as VLCAD deficiency in itself is a rare metabolic disease, and *FBXL4* -related encephalomyopathy MTDPS represents an even rarer occurrence. Two different gene variants were found: a homozygous FBXL4 variant c.1698A > G and an ACADVL variant c.134C > A. The occurrence of these variants in isolation have been described, but, to the best of our knowledge, both these gene variants occurring together in one patient to cause two different metabolic diseases have not yet been described in the literature.

A defect of FBXL4 protein function results from FBXL4 gene variants that cause FBXL4-related encephalomyopathic MTDPS13. It is known as mtDNA depletion when there are abnormalities in the maintenance of mtDNA as a result of lack activity of this protein. Many of the body cells have impaired mitochondrial activity as a result of mtDNA depletion. Cell dysfunction brought on by diminished mitochondrial function eventually manifests most visibly in the brain, muscles, and other tissues with high energy requirements. Encephalomyopathy and other symptoms of the FBXL4-related encephalomyopathic MTDPS are caused by this cell malfunction.

Most of the affected patients with *FBXL4* -related encephalomyopathic **MTDPS13** usually present with global developmental delay, hypotonia, and persistent lactic acidosis resulting in early death, as Bonnen et al 2 reported in three unrelated consanguineous families. Microcephaly, craniofacial abnormalities, and congenital cataracts have also been documented. Gai et al 4 also reported nine children with early-onset mitochondrial encephalomyopathy. Most of them presented with lactic acidosis, neutropenia, and hyperammonemia. All the patients had severe psychomotor retardation, hypotonia, seizures, and failure to thrive, and reported three infant deaths from metabolic decompensation related to a severe illness. Our patient had clinical phenotypes similar to those of the patients reported in these studies, but he did not show hyperammonemia or seizures. During the physical examinations, most of the previous patients showed scoliosis, small feet, hypospadias, and dysmorphic features. Facial features included microcephaly, malformed protruding ears, cataracts, a narrow, elongated face, thick eyebrows, epicanthal folds, downslanting of the space between the eyelids, and short, upturned nose. Unfortunately, magnetic resonance imaging (MRI) of the brain was not done due to the patient's critical and unstable condition, but the majority of brain MRI scans done in previous cases have demonstrated generalized cerebral atrophy, cerebellar hypoplasia, dilated ventricles, a thin corpus callosum, and altered signals in the supratentorial and infratentorial white matter with involvement of the basal ganglia and thalami. An echocardiogram documented hypertrophic cardiomyopathy in two patients with encephalomyopathy due to MTDPS13. Therefore, the involvement of hypertrophic cardiomyopathy in our patient can be due to VLCAD deficiency by itself, MTDPS13, or both. The FBXL4 variant c.1698A > G p. (Ile566Met) causes an amino acid change from Ile to Met at position 566. According to HGMD Professional 2022.1, this variant has previously been described as a disease causing developmental delay and lactic acidosis. 3 6 7

In our patient, recurrent hypoglycemia was the most clinical sign of a metabolic disease. A lengthy sequence of biochemical and genetic investigations led to the identification of VLCAD deficiency as a second disease, explaining this “challenging” phenotype in inherited metabolic diseases. 8

Even if the diagnosis requires ACADVL genetic analysis and/or functional tests on fibroblasts or lymphocytes, the development of newborn screening programs has increased the frequency of VLCAD cases. ACADVL has so far been reported to have more than 300 known variants ( http://www.hgmd.cf.ac.uk/ac/gene.php?gene=ACADVL ). The herein-described patient carried the ACADVL variant c.134C > A p. (Ser45*), which creates a premature stop codon. According to HGMD Professional 2022.1, this variant has previously been described as causing a VLCAD deficiency. 7

The most prevalent clinical phenotype for VLCAD deficiency is a severe early-onset variant of VLCAD associated with significant mortality and high incidence of cardiomyopathy. Compared with encephalomyopathic MTDPS13, an uncommon cardiomyopathy has been reported in only two patients so far. 4 The outcome of VLCAD deficiency in Saudi Arabia remains poor, with recurrent admission for metabolic crisis despite early diagnosis detected by newborn screening and immediate treatment with a special formula containing medium chain triglyceride (MCT) and carnitine. So preventive methods like prenatal diagnosis and preimplantation genetic diagnosis are considered important to reduce the incidence of inherited diseases, 9 especially the incidence of more than just metabolic disorders, which is the most probable explanation for the high rate of consanguinity in the Saudi Arabian population.

## Conclusion

In this particular infant, the occurrence of these two uncommon metabolic disorders resulted in quite an unusual clinical presentation. The rise in “double trouble” instances shows that it is worthwhile to look for a more accurate diagnosis when an already established phenotype is compounded by unusual traits and difficult diagnosis.
